# Conformational Dynamics of Human ALKBH2 Dioxygenase in the Course of DNA Repair as Revealed by Stopped-Flow Fluorescence Spectroscopy

**DOI:** 10.3390/molecules27154960

**Published:** 2022-08-04

**Authors:** Lyubov Yu. Kanazhevskaya, Denis A. Smyshliaev, Nadezhda A. Timofeyeva, Alexander A. Ishchenko, Murat Saparbaev, Nikita A. Kuznetsov, Olga S. Fedorova

**Affiliations:** 1 Institute of Chemical Biology and Fundamental Medicine (ICBFM), 8 Lavrentiev Ave., 630090 Novosibirsk, Russia; 2 Department of Natural Sciences, Novosibirsk State University, 1 Pirogova St., 630090 Novosibirsk, Russia; 3 Group “Mechanisms of DNA Repair and Carcinogenesis”, Gustave Roussy Cancer Campus, CNRS UMR9019, Université Paris-Saclay, 94805 Villejuif, France

**Keywords:** DNA repair, dioxygenase ALKBH2, DNA methylation, conformational dynamics, fluorescent spectroscopy, pre-steady-state kinetics, stopped-flow, aminopurine, FRET analysis

## Abstract

Elucidation of physicochemical mechanisms of enzymatic processes is one of the main tasks of modern biology. High efficiency and selectivity of enzymatic catalysis are mostly ensured by conformational dynamics of enzymes and substrates. Here, we applied a stopped-flow kinetic analysis based on fluorescent spectroscopy to investigate mechanisms of conformational transformations during the removal of alkylated bases from DNA by ALKBH2, a human homolog of *Escherichia coli* AlkB dioxygenase. This enzyme protects genomic DNA against various alkyl lesions through a sophisticated catalytic mechanism supported by a cofactor (Fe(II)), a cosubstrate (2-oxoglutarate), and O_2_. We present here a comparative study of conformational dynamics in complexes of the ALKBH2 protein with double-stranded DNA substrates containing N1-methyladenine, N3-methylcytosine, or 1,N6-ethenoadenine. By means of fluorescent labels of different types, simultaneous detection of conformational transitions in the protein globule and DNA substrate molecule was performed. Fitting of the kinetic curves by a nonlinear-regression method yielded a molecular mechanism and rate constants of its individual steps. The results shed light on overall conformational dynamics of ALKBH2 and damaged DNA during the catalytic cycle.

## 1. Introduction

Fe(II)/α-ketoglutarate–dependent dioxygenases of the AlkB subfamily play a crucial role in direct reversal repair of small alkyl lesions in DNA and RNA nucleobases [1,2]. These proteins belong to the superfamily of nonheme iron enzymes catalyzing oxidation of bioorganic substrates by molecular oxygen (O_2_) [3]. The reaction mechanism established for these enzymes proceeds via oxidative decarboxylation of a cosubstrate: 2-oxoglutarate. A highly reactive “oxyferryl” enzyme-bound intermediate, Fe(IV) = O, is responsible for the hydroxylation of alkylated nucleobases [4]. The resultant hydroxylated hemiaminal adduct is spontaneously hydrolyzed, generating the intact base. Among various mammalian AlkB orthologs, the ALKBH2 protein is a major dioxygenase for the repair of alkyl lesions in genomic DNA, especially in ribosomal-DNA genes [5,6]. Broad substrate specificity of ALKBH2 covers N1-, N3-alkyl, and exocyclic bridged adducts of purine and pyrimidine nucleotides in double-stranded DNA (dsDNA) [7,8,9,10]. Effective repairing of 1,N6-ethenoadenine by ALKBH2 on occluded sites of the nucleosome core particle was recently reported [11]. Altered expression levels of this dioxygenase in various human malignant tumors are indicative of its biological importance [12,13,14]. Downregulation of ALKBH2 in human cancers is among pieces of evidence supporting its possible clinical significance, as is the ability to increase sensitivity of cancer cells to chemotherapy [15,16].

**Figure 1:**
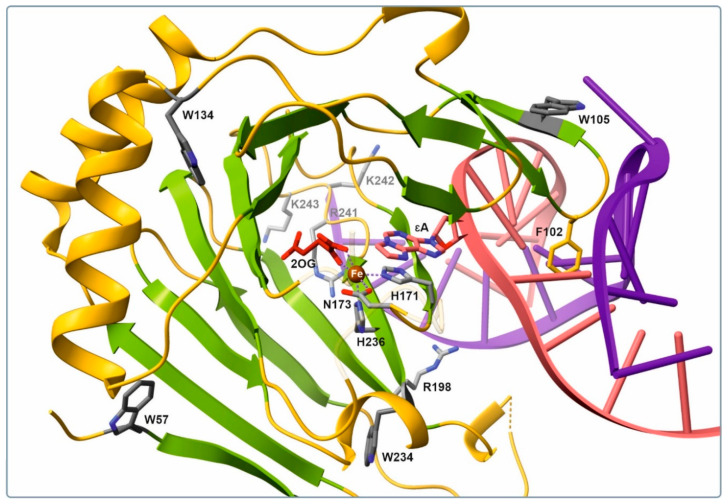
Closeup view of the active site of ALKBH2 with Fe (brown), 2OG (red), and εA-containing dsDNA (pink/violet) according to Protein Data Bank structure 3RZK [18]. Key coordinating and intercalating amino acid residues are indicated. For clarity, two protein regions (aa 174–179 and 256–258) are shown as 90% transparent cartoons.

ALKBH2 shares a conserved double-stranded β-helix (DSBH) structural fold and employs a reaction mechanism common among other 2-oxoglutarate (2OG) dependent dioxygenases (Figure 1) [17,18,19]. The sequence of ALKBH2 comprises a poorly ordered N-terminal domain, the DSBH catalytic core, and a nucleotide recognition lid (NRL motif) connected by specific loop regions [20]. The Fe(II) ion is coordinated by highly conserved triad 2-His–1-Asp (His-171, Asp-173, and His-236), a bidentate 2OG, and a water molecule that is displaced by O_2_ after DNA substrate binding. ALKBH2 binds to the damaged DNA strand via a short positively charged RKK (Arg-241, Lys-242, and Lys-243) loop and holds the complementary strand using a long loop motif containing residues Arg198, Gly204, and Lys205. The process of lesion recognition, binding, and oxidation requires flipping of an alkylated base into the enzyme active site pocket. It has been shown that individual structural peculiarities inherent in enzymes EcAlkB (*Escherichia coli* AlkB) and ALKBH_i_ underlie a substantial difference in their substrate specificity and biological functions. In particular, ALKBH2 uses a unique mechanism to flip and retain the damaged base: insertion of a finger residue Phe-102 from a β3–β4 hairpin into the duplex stack and filling the space resulting from the base flipping [17]. In contrast to EcAlkB, the binding of dsDNA by the ALKBH2 protein induces a little distortion/bending of the substrate duplex [18,21]. A similar dual β-hairpin wedge has been found in AAG DNA glycosylase [22]. Of note, the important β3–β4 hairpin in ALKBH2 (amino acid residues [aa] 89–108) contains predominantly hydrophobic residues, in contrast to charged and hydrophilic residues in ALKBH3. It is believed that this divergent β-hairpin forms a unique functionally important hydrophobic network in the ALKBH2 active site, ensuring preferences for double-stranded substrates [23,24]. Other DNA-binding loops of ALKBH2 are also different from the corresponding loop motifs in ALKBH homologs [20]. All of the aforementioned features offer great diversity of catalytic-cleft size, conformational mobility, and other functional characteristics of AlkB family dioxygenases. In our recent study, we attempted to probe single-turnover conformational dynamics of EcAlkB in the course of damaged-DNA repair by stopped-flow fluorescent spectroscopy [25].

ALKBH2 is highly active against alkyl lesions in dsDNA and interacts poorly with single-stranded DNA. It is assumed that this property is due to its structural features, in particular its longer DNA-binding loops, which are distinct from loop motifs of other AlkB family enzymes. To date, the structure of ALKBH2 and its complex with damaged DNA have been largely based on static X-ray crystallography data. At the same time, there is a need for information on changes in the structure of the enzyme–substrate complex in solution during the catalytic cycle. There is evidence that conformational flexibility of ALKBH2 and DNA substrate domains is important for their effective interaction [18,21]. These notions are mainly based on crystallographic and quantum mechanics/molecular mechanics (QM/MM) modeling data, and further studies are necessary to experimentally detect conformational rearrangements in the catalytic complex under physiological conditions. A biophysical stopped-flow (SF) approach combined with fluorescence spectroscopy can serve as a convenient tool for examining conformational changes of the enzyme and DNA substrate in real time [26].

This study is the first attempt to directly detect conformational transitions in the complex of ALKBH2 with alkylated DNA by fluorescence spectroscopy. Via rapid mixing of the enzyme and its substrate in a stopped-flow apparatus, changes in the conformation of the enzyme and DNA substrates were detected at individual steps of the catalytic cycle. Own fluorescence of the reacting moieties (Trp and 1,N6-ethenoadenine [εA]) was measured, as was the fluorescence of the labels introduced into appropriate positions of DNA substrates (aminopurine (aPu) and FAM-BHQ1). Taken together, the obtained data on conformational transitions should elucidate the kinetic model of induced tuning (of the enzyme–substrate complex) intended to achieve a catalytically active state.

## 2. Results

### 2.1. Equilibrium Binding of ALKBH2 to Metal Ions, Cosubstrate, and Methylated DNA

**Figure 2:**
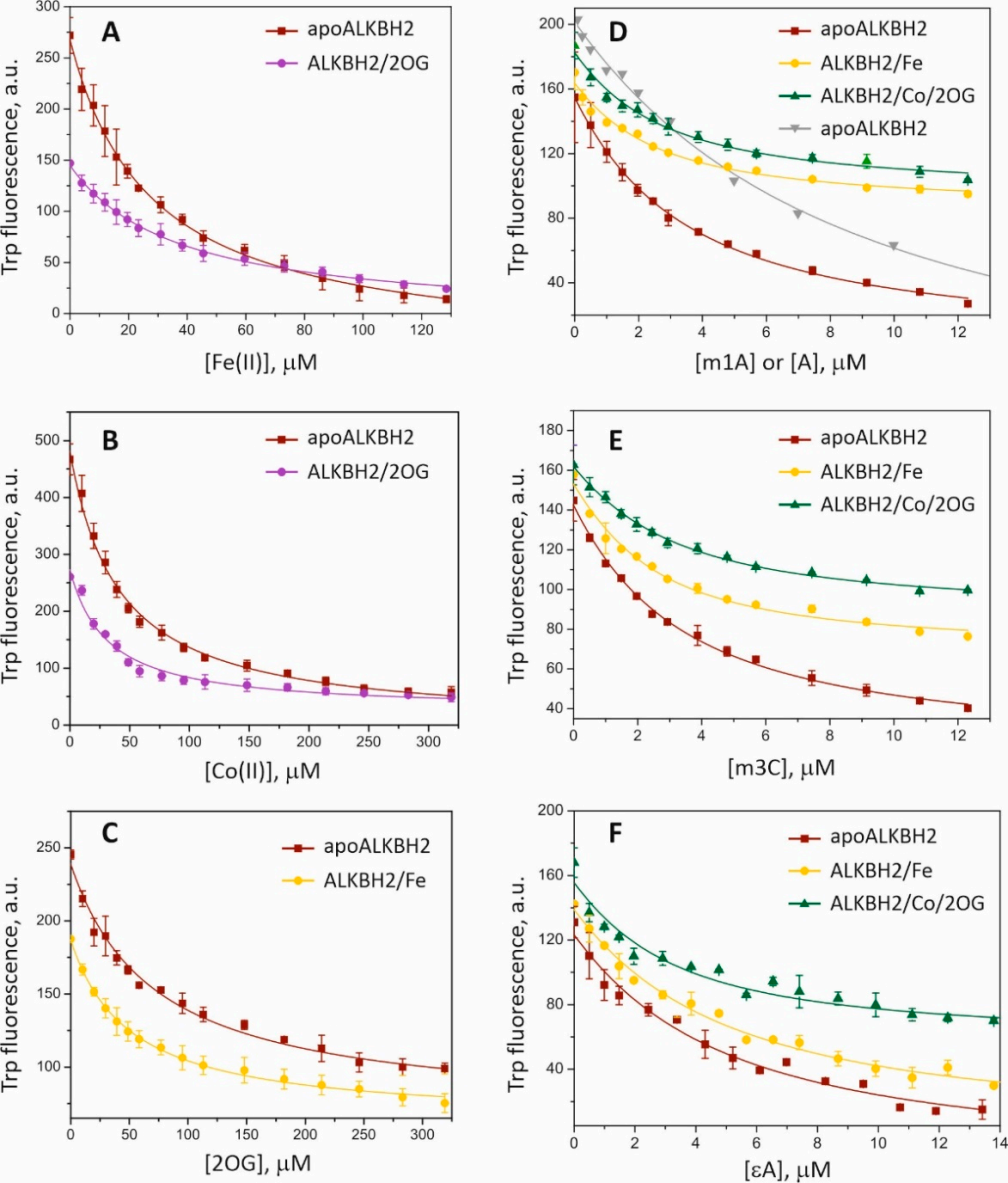
Equilibrium binding of ALKBH2 to metal ions, 2OG, or damaged DNA. Trp fluorescence of the enzyme is shown as a function of concentration of a metal ion (**A**,**B**), 2OG (**C**), or DNA substrate (**D**–**F**). Gray triangles in panel D indicate titration of the ALKBH2/Fe(II)/2OG complex with a 15 nt undamaged ODN containing A instead of m1A. The enzyme concentration was 1 μM. The concentrations of Fe(II), Co(II), and 2OG within preformed ALKBH2/Me(II) or ALKBH2/Me(II)/2OG complexes were 40 μM, 100 μM, and 1 mM, respectively. Fluorescent titration of apoALKBH2 was carried out in the presence of 2 mM EDTA. Experimental data points were fitted to a single-site binding model. Each trace represents the average of three independent assays. In the figures, for better presentation, the curves were manually moved apart.

**Figure 3:**
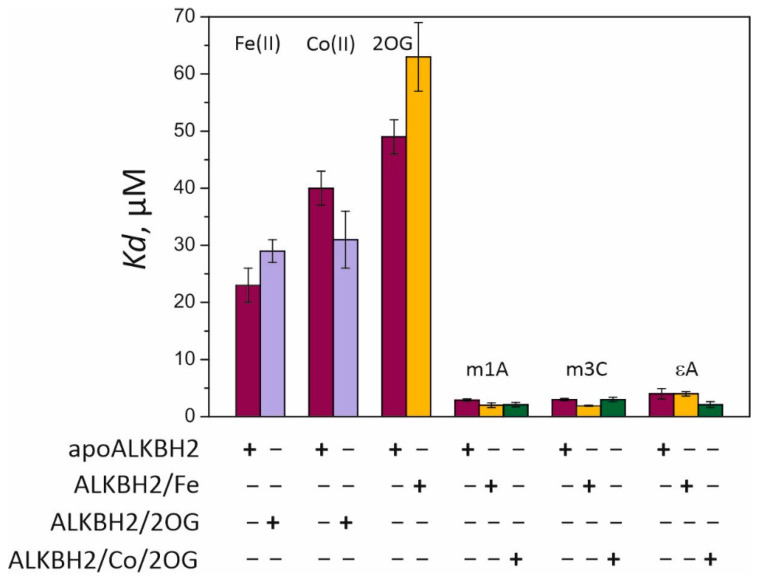
Analysis of ALKBH2 binding affinity by the equilibrium titration assay. The enzyme was titrated with metal ions (Fe(II) or Co(II)), 2OG, and methylated DNA at 25 °C. Dissociation constants (*K_d_*) were determined by the fitting of experimental data points to a quadratic binding equation (Equation (2); see Section 4.3). The efficiency of ligand binding to apoALKBH2 is presented as wine-colored bars. *K_d_* for the titration of preformed complexes ALKBH2/Fe, ALKBH2/2OG, and ALKBH2/Co/2OG is presented in lilac, yellow, and green colors, respectively. Error bars represent standard deviation of three technical replicates.

To assess the affinity of ALKBH2 for its cofactor, to a cosubstrate, and to methylated and nonmethylated DNA (Table 1), Trp fluorescence of the protein was monitored upon titration with the ligands (Figure 2). It was found that equilibrium dissociation constant (*K_d_*) values for complexes of the free enzyme (apoALKBH2) with Fe(II) and Co(II) are 27 and 40 μM, respectively (Figure 3). Thus, Co(II) can compete with Fe(II) for the iron-binding site only at concentrations twofold lower than those observed for Ni(II) [27]. Incubation of ALKBH2 with 2OG prior to the titration with metal ions slightly reduced binding affinity for Fe(II) and increased affinity for Co(II). The lowest stability was observed for the complex of ALKBH2 with its cosubstrate 2OG, while preincubation of the protein with Fe(II) did not improve but rather reduced the binding efficiency of 2OG. In our previous work [28], we studied an ability of the EcAlkB protein to bind different metal cofactors, a cosubstrate, and methylated DNA. Those results indicated that EcAlkB has 2–4 times higher affinity for Fe(II) and 2OG than for Co(II), and the formation of the complex between the dioxygenase and 2OG is not affected by preincubation with a metal ion.

It is known that transition metals other than Fe(II) inhibit the catalytic activity of AlkB-like proteins while maintaining their ability to bind DNA substrates [17,27,29,30]. Here, we determined the binding affinity of ALKBH2 toward alkylated DNA in the presence of Co(II) ions. ApoALKBH2 and preformed complexes ALKBH2/Fe, ALKBH2/Co/2OG (1 μM) were titrated with 15 nt dsODN containing N1-methyladenine (m1A), N3-methylcytosine (m3C), or εA. *K_d_* values for methylated-DNA binding were calculated by means of Equation (2) (see Section 4.3 for details). They are in the range of 1.9–4.0 μM, which is one order of magnitude lower than that of metal ions and 2OG, indicating higher affinity of ALKBH2 for the DNA substrate than for its cofactor and cosubstrate. Similar affinity patterns have been observed during fluorescent titration of the EcAlkB protein [28,29]. Incubation of ALKBH2 with the Fe(II) ion prior to the titration resulted in a 1.5-fold increase in the affinity for the m1A- and m3C-containing substrates but did not affect the efficiency of binding to the εA-containing substrate. In a comparison of the efficiency of DNA binding to preformed complex ALKBH2/Co/2OG vs. apoALKBH2, the most pronounced effect was observed in the case of the εA-containing substrate accompanied by a 2-fold reduction in *K_d_*. As a control, we also measured Trp fluorescence quenching upon titration of ALKBH2 with an intact 15 nt dsODN (Figure 2D, gray triangles). The resultant *K_d_* reached 7.8 ± 1.2 μM, which meant that ALKBH2 binds to alkylated DNA in a more specific manner. Overall, the fluorescence spectroscopy data indicated that the binding of damaged DNA by the ALKBH2 protein is preferable over the binding to the metal cofactor and cosubstrate.

### 2.2. ALKBH2 Repair Activity toward DNA Lesions m1A, m3C, and εA

**Figure 4:**
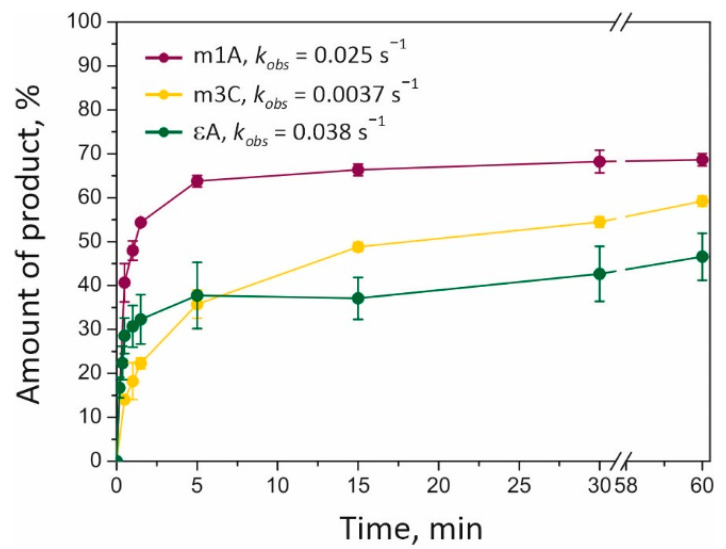
ALKBH2-catalyzed repair of m1A-, m3C-, and εA-containing dsODNs. Time courses of the dealkylated-product accumulation were obtained at 25 °C by mixing of 2 μM enzyme with 2 μM FAM-labeled dsDNA substrate dissolved in reaction buffer containing 40 μM Fe (II) and 1 mM 2OG (see Section 4). Each reaction time point is the mean ± SD of three technical replicates.

Activity of the recombinant human ALKBH2 protein toward damaged dsDNA substrates was measured by a restriction enzyme-coupled assay followed by gel electrophoresis digestion. The reaction mixture contained equimolar amounts of the enzyme and its substrate preincubated with Fe(II) and 2OG. After termination of the reaction at each time point, samples were treated with a specific restriction endonuclease to cleave the intact DNA product (see Section 4.4 for details). A shortened reaction product was next visualized on a denaturing polyacrylamide gel, revealing time courses of the substrate dealkylation (Figure 4). The amount of dealkylated DNA varied from 40 to 70% depending on the lesion type (m1A > m3C > εA). Repair of the m1A base is carried out somewhat more slowly but more efficiently than the repair of the εA base is. The lowest rate of repair (with a medium amount of the product: ~58% after 2 h) was detected in the case of the m3C-containing substrate (m3C). Our data indicated that m1A is the most effective substrate for ALKBH2 among those analyzed. 

The observed rate constant (*k_obs_*; see Section 4.4 for details) of dealkylation reactions depended on the lesion as follows: εA (0.038 s^−1^) > m1A (0.025 s^−1^) > m3C (0.0037 s^−1^). Our data suggested that an initial reaction rate does not correlate with the maximum amount of dealkylated product formed for each lesion. The highest reaction rate was documented for the repair of the εA-containing substrate, which also gave the lowest amount of the product. The presence of fast and slow phases of product accumulation in the case of εA base oxidation may be attributed to a tight binding of the reaction product within the ALKBH2 active site; this event impairs dissociation of the enzyme–product complex. This hypothesis can find support in previous findings about EcAlkB and ALKBH2. As shown by NMR spectroscopy, the EcAlkB/Fe/Suc complex has higher flexibility as compared to the 2OG-bound complex, and this property is believed to be important for an effective product release [31]. On the other hand, recent data obtained by the QM/MM method indicate that the ALKBH2/dsDNA complex is more compact and rigid (than the EcAlkB/dsDNA complex), and these properties may result in tighter binding of the product [21]. Thus, slow dissociation of the enzyme–product complex can inhibit the activity on the next enzymatic cycle.

So far, no data have been published that describe in vitro kinetic parameters for both methylated and etheno lesions under the same conditions (reagent amounts, temperature, and a nucleotide context). Two research groups have investigated Michaelis–Menten kinetics of ALKBH2 toward m1A- and m3C-containing DNA and reported markedly distinct values of *k*_cat_ and *K*_M_ [8,32]. For example, *k*_cat_ for lesion m3C within a 24 nt dsODN was found to be 8.8 s^−1^ [8], whereas *k*_cat_ for the same lesion within a 16 nt dsODN was 0.043 s^−1^ [32]. As for lesion εA, no steady-state kinetics of its hydroxylation by ALKBH2 have been reported at all. Single-turnover kinetics measured by Ringvoll et al. [10] revealed the efficiency of εA removal of 0.094 min^−1^ in terms of the turnover number, and the largest amount of the dealkylated product did not exceed 15%. Another study suggests that the specific repair activity of ALKBH2 against εA is 69.4 fmol min^−1^ pmol enzyme^−1^, with 70% of the repaired dsODN detected after 10 min of incubation [9]. The existence of such fragmentary or controversial data may be due to variation of the proportion of an active fraction in enzyme samples among studies. We detected a similar problem in the course of the protein purification; therefore, all experiments were conducted on the same ALKBH2 sample that manifested the highest activity level.

### 2.3. SF Fluorescence Measurements of Conformational Changes in the Complex of ALKBH2 with a Monoalkyl Lesion (m1A or m3C)

**Figure 5:**
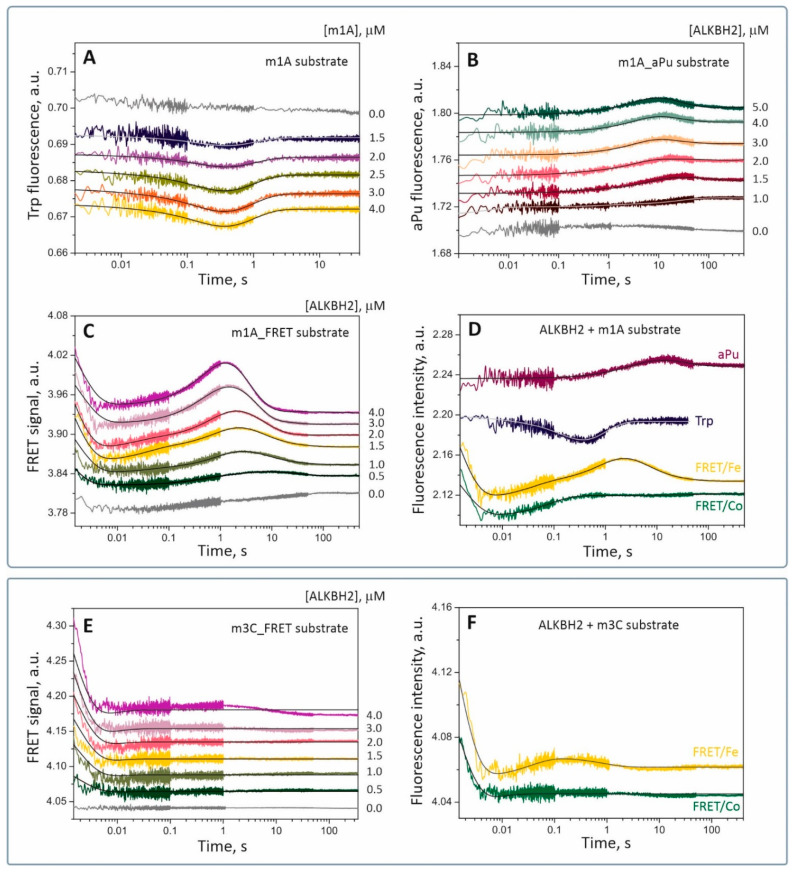
Interactions of ALKBH2 with m1A- and m3C-containing DNA as measured by the SF method. (**A**) Time courses of Trp fluorescence intensity obtained for 2 μM enzyme and various concentrations of substrate m1A. (**B**) Time courses of aPu fluorescence intensity obtained for 2 μM aPu-containing substrate m1A and various concentrations of ALKBH2. (**C**) Time courses of the FRET signal generated by the FAM/BHQ1 pair in the presence of 1.5 μM DNA substrate and various concentrations of ALKBH2. Gray curves represent control experiments where the fluorescent component was mixed with the buffer. (**D**) A comparison of SF analysis-derived kinetic curves for different fluorophores and metals in the course of demethylation of substrate m1A. Experimental data on Trp, aPu, and FRET are highlighted in dark blue, wine, and yellow, respectively. The green curve was obtained during interactions of ALKBH2 and a methylated substrate in the presence of Co(II) as a cofactor. (**E**) Time courses of the FRET signal obtained for 1.5 μM substrate m3C and various concentrations of ALKBH2. (**F**) A comparison of FRET kinetic curves between cofactors Fe(II) and Co(II) as measured in the course of interactions between ALKBH2 and substrate m3C. For each set of curves, jagged traces represent experimental data. Smoothed curves were obtained via global fitting to kinetic Scheme 1 and Scheme 2.

To investigate in detail the changes in the conformation of the enzyme–substrate complex in the course of DNA dealkylation, we measured a fluorescent signal from various fluorescent probes sensitive to changes in their local environment. Equimolar amounts of ALKBH2 and a model DNA substrate containing a lesion (m1A or m3C) were mixed in an SF spectrometer cell in the presence of Fe(II) and 2OG. Time-dependent conformational changes of the protein were recorded as changes in the fluorescence of its Trp residues. Detection of the conformational transitions in the DNA substrate structure was carried out using two fluorophores. The first one was a fluorescent analog of adenine aPu introduced into the damaged strand 3′ to the m1A base, and the second was a dye-quencher pair FAM-BHQ1 located at the 5′ end and 3′ end of the damaged strand, respectively, for measuring fluorescence resonance energy transfer (FRET; see Table 1). According to preliminary experiments, interaction of ALKBH2 with the substrate containing the m1A lesion caused significant changes in the local environment of each fluorescent probe, whereas binding to an m3C-containing substrate had very little effect on fluorescence intensity of the labels. Further SF analysis was performed with fluorophores sensitive to changes in the local environment. A series of SF traces was obtained for each substrate under approximately single-turnover conditions for a fixed concentration of the fluorescent component (1.5 or 2.0 μM) and various concentrations of its nonfluorescent counterpart (0.5 to 5.0 μM; Figure 5). In the case of substrate m3C, we were able to detect a concentration-dependent change in the FRET signal. In each case, a control curve was obtained by mixing the fluorescent component with reaction buffer to avoid detection of nonspecific changes in the signal (Figure 5, gray curves).

It was found that conformational dynamics of the ALKBH2 protein during interactions with substrate m1A are biphasic (Figure 5A). In the first phase, Trp fluorescence is quenched (up to time point 300 ms), and then it gradually grows in the interval 0.3 s to 5 s. By analyzing available X-ray structural data [17,18], we attempted to estimate the possible contribution of each of the four Trp residues of ALKBH2 (see Figure 1) to the overall signal detected by the SF analysis. A side chain of Trp-105 proved to be the most important contributor because it belongs to the aromatic β3–β4 hairpin, which interacts closely with the damaged strand of the substrate duplex. This finger motif participated in capture of the substrate via a side chain of Phe-102, which occupies the vacant space created by methylated-base flipping. Other residues (Trp-57, -134, and -234) are likely to make only a minor contribution to the changes in fluorescence intensity because they are located on the protein surface far from the active site. Therefore, conformational changes of the enzyme observed by the SF method may represent fluctuations in the Trp-105 microenvironment in the course of embedding of the specific loop into the DNA substrate groove.

Behavior of the aPu fluorescent probe located adjacently to the m1A base showed no specific changes in the local environment of the fluorophore immediately after mixing with ALKBH2 (Figure 5B). Nonetheless, starting from 400 ms, the aPu fluorescent signal underwent noticeable growth and reached its maximum in the range 10 s to 30 s, depending on the enzyme concentration. Next, there was a moderate decrease in fluorescence intensity up to 100 s. The fluorescent properties of aPu are described in the literature [33,34]. In particular, the fluorescence of aPu is quenched in solution by a hydrophobic environment and is enhanced after the transition to a more hydrophilic medium. Thus, we can assume that the interaction of enzyme ALKBH2 with substrate m1A in the middle and late stages of the catalytic cycle is accompanied by a bending of the DNA duplex in the region of the damaged nucleotide, as evidenced by an increase in solvent polarity around the aPu.

Analyses of protein–DNA interactions on the basis of the FRET phenomenon are widely used in biochemistry [35]. Interactions between the enzyme and FRET-labeled damaged DNA cause a bending of the substrate duplex, resulting in fluctuation of the distance between the donor and acceptor and changes in FRET efficiency. Therefore, by measuring the intensity of the fluorescence transfer from the donor dye, we can detect protein-induced conformational changes in the DNA substrate with high accuracy. Here, we measured the FRET signal from the 5′-FAM/3′-BHQ1 pair during interactions of ALKBH2 with substrate m1A or m3C. As follows from Figure 5C,E, the interaction of ALKBH2 with the FRET-labeled substrate caused multiple changes in the signal. The initial parts of the FRET curves for both substrates contain a sharp decrease in the signal from 1 ms to 5 ms, pointing to a spatial convergence of the fluorophore and quencher, and may mean the bending of the DNA helix when it binds to the dioxygenase. This conformational transition in the substrate molecule most likely accompanies the formation of a nonspecific collision complex. The next stage of substrate m1A processing was followed by a pronounced two-phase growth of the FRET signal with a peak at 80 ms and 1.5–2.0 s, respectively. These transitions denote spatial separation of FAM and BHQ1 and likely correspond to fine tuning of the nucleoprotein complex toward a catalytically active state. As for substrate m3C, low-amplitude alterations of the signal were detected after 10 ms on FRET curves corresponding to high ALKBH2 concentrations. A possible interpretation of this type of signal change is weak or slow conformational rearrangements in catalytic complex ALKBH2/m3C.

Some structural studies on AlkB family proteins have shown that bacterial and human 2OG dioxygenases can bind ions of transition metals other than Fe(II) (e.g., Co(II), Ni(II), Mn(II) or Cu(II)) with high affinity while retaining the proper tertiary structure [17,36,37,38]. At the same time, replacing Fe(II) with other metal ions leads to complete inhibition of the dealkylating activity [27,30]. This phenomenon can be exploited for the detection of substrate recognition and binding processes separately from hydroxylation by the SF method.

In the present study, we implemented this strategy by replacing Fe(II) with the Co(II) ion at the ALKBH2 active site. As we demonstrated by fluorescent titration (Section 2.1, Figure 2B and Figure 3), cobalt binds to the ALKBH2 active site with the same efficiency as iron does in the presence of 2OG. To elucidate conformational dynamics of the enzyme–substrate complex at steps preceding the catalysis, 1.5 μM ALKBH2 was mixed with 1.5 μM substrate m1A or m3C in the presence of 100 μM CoSO_4_. Figure 5D,F depict time courses of the FRET signal (green curves) registered by the SF method under conditions similar to the Fe-containing system. In a comparison of FRET curves obtained in the presence of Fe(II) (yellow curves) and Co(II) ions, one can see that shapes of the curves matched in the interval 1–400 ms for substrate m1A and 1–30 ms for substrate m3C. After this period, no specific changes in fluorescence were noted on the curves in the presence of Co(II). Subsequent changes in the signal detected in the presence of Fe(II) most probably correspond to specific transitions that require the presence of a natural cofactor. For the ALKBH2/Co(II)/2OG/m3C complex, the FRET curve contained a single conformational transition (in the interval 1–20 ms) similar to that of the ALKBH2/Fe(II)/2OG/m3C complex (Figure 5F). Consequently, replacement of the natural metal-cofactor with Co(II) ion does not affect the initial binding but complicates subsequent transformation of the enzyme–substrate complex.

### 2.4. Assessment of ALKBH2 Conformational Dynamics and Kinetic Parameters for the m1A- and m3C-Containing dsODNs

To quantitatively describe and interpret the observed conformational transitions in the light of specific stages of the molecular-kinetic mechanism, data obtained under similar conditions by different methods were analyzed. In particular, the fluorescent curves determined by the SF method were compared with time courses of reaction product accumulation revealed by polyacrylamide gel electrophoresis (PAGE). Half-conversion time (τ_1/2_) of substrate m1A (Figure 4), as determined by means of ratio ln2/*k_obs_*, is approximately 30 s. This time matches the phase of the decrease in aPu fluorescence intensity between time points 20 and 80 s (Figure 5B) and overlaps with the phase of a FRET signal reduction between 2 s and 50 s (Figure 5C). Accordingly, these two conformational transitions of substrate m1A may accompany the steps of its hydroxylation and dissociation of the enzyme–product complex. As follows from PAGE analysis of ALKBH2 repair activity (Figure 4), hydroxylation of substrate m3C takes much more time (τ_1/2_ ≈ 180 s) as compared to substrate m1A. Therefore, the changes in substrate m3C′s conformation as recorded by FRET (Figure 5E) may reflect the binding process at the ALKBH2 active site and DNA duplex adjustment on the way to the catalytically active complex but do not provide information about the catalytic step.

To compare the conformational transitions observed using different fluorophores, one curve with clearly distinguishable conformational transitions was selected from each kinetic series, and individual phases were compared qualitatively (Figure 5D,F). This approach revealed that the initial binding of ALKBH2 to substrate m1A (up to time point ~8–10 ms) does not cause significant alterations of protein conformation in the region of intercalating loop β3–β4 (no change in Trp fluorescence). The DNA duplex undergoes some bending, which brings its ends closer together, but does not affect solvent polarity near the damaged nucleotide (no change in aPu fluorescence). Once the enzyme has detected the presence of a methylated base in the DNA substrate, its conformation changes, and a specific interaction with the substrate begins, as evidenced by synchronous quenching of Trp fluorescence (embedding of the intercalating loop into the DNA groove) and an increase in the FRET signal (eversion of the damaged nucleotide into the active site pocket) (up to time point ~100 ms). As a result, the aPu residue adjacent to m1A becomes more accessible to the solvent, and its fluorescence begins to increase gradually (up to ~10 s). At the same time, conformational transitions in the catalytic complex continue, accompanied by a more pronounced increase and a subsequent decrease in the FRET signal as well as a return of Trp fluorescence to the initial level. By comparing all of the data, we gained important knowledge that allowed us to correlate the observed conformational transitions with individual phases of the catalytic cycle. For example, the first two phases of FRET signal changes appeared to be associated with the formation of a specific complex between ALKBH2 and substrate m1A.

**Scheme 1:**
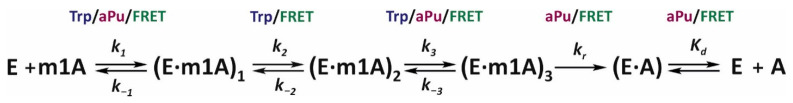
The kinetic mechanism of m1A lesion demethylation by ALKBH2 according to the SF data (Figure 5A–C). E is the enzyme coordinated with Fe(II) and 2OG; m1A is the DNA substrate containing m1A; A is a repaired DNA product; (E∙m1A)_i_ is an intermediate enzyme–substrate complex. Rate constants *k_i_* and *k_−i_* (*i* = 1, 2, and 3) characterize forward and reverse directions of the equilibria corresponding to the steps of substrate binding and adjusting for catalysis. Rate constant *k_r_* describes the irreversible step of substrate hydroxylation; *K_d_* is an equilibrium constant of enzyme–product complex (E∙A) dissociation and is calculated from the DNA conformational dynamics. The fluorescent probes used for the detection of individual steps are highlighted above the arrows designating the reaction steps.

To choose an appropriate kinetic scheme and to determine the kinetic parameters, the DynaFit 4 software was employed [39]. During the selection of the kinetic scheme, its sequential complication was carried out, and deviations of the theoretical curves from the experimental ones were evaluated. The rate constants for each elementary step were obtained via optimization of the parameters during numerical integration of the system of differential equations [25,40]. As a result, it was determined that the lowest number of steps needed to describe the experimental curves for the demethylation of substrate m1A is five. These are three reversible steps described by rate constants *k_i_* and *k_−i_* (*i* = 1, 2, and 3) and one irreversible step (*k_r_*), followed by the decay of the enzyme–product complex (*K_d_*) (Scheme 1). Thus, the sequence of changes in the conformation of the enzyme–substrate complex can be described as follows. The initial binding of ALKBH2 to a DNA duplex leads to the formation of a nonspecific complex (E∙m1A)_1_, which isomerizes successively into specific (E∙m1A)_2_ and catalytically competent (E∙m1A)_3_ complexes. This event is followed by a step of irreversible hydroxylation of the substrate to form the enzyme–product complex (E∙A) and its dissociation. It is important to note that not every stage of the mechanism could be detected by each of the three fluorophores. Particularly, Trp fluorescence did not provide information about the dynamics of the ALKBH2 conformation at the substrate hydroxylation step, and aPu fluorescence failed to sense the second stage of isomerization of the initial complex.

In Table 2, the rate constants corresponding to each reaction of Scheme 1 are summarized. The *k*_1_ constants derived by means of different fluorophores suggest that the formation of complex (E∙m1A)_1_^FRET^ (detected by FRET) precedes the formation of the complex (E∙m1A)_1_^Trp^ and (E∙m1A)_1_^aPu^ (detected via fluorescence intensities of Trp and aPu), respectively. This means that recognition and binding of DNA by ALKBH2 first leads to an alteration in the conformation of the substrate and only then causes changes in the local environment of the protein Trp residues. If one assumes that the main contribution to the fluorescence of the enzyme is made by residue Trp-105, then the observed changes in protein conformation should be associated with the dynamics of the β3–β4 hairpin, which interacts with the damaged DNA strand to retain the substrate. Consequently, we can theorize that damage recognition within the DNA duplex begins with its bending by the enzyme. The second conformational transition in the structure of the enzyme–substrate complex is characterized by forward rate constant *k*_2_ values 7.2 and 10 s^−1^ for the Trp fluorescence and FRET signal, respectively. Given that this difference is statistically insignificant, these constants may be describing the same step of the mechanism. On the other hand, this conformational transition should proceed more efficiently in the protein (*k*_−2_^Trp^ = 3.2 s^−1^) than in the substrate (*k*_−2_^FRET^ = 21 s^−1^). The third reversible step in the kinetic scheme is described by the *k*_3_ values in the range of 0.67–2.2 s^−1^, with the lowest value found for aPu, which shows the long increase in the signal. Taking into account fluorescent properties of aPu, we supposed that this base becomes more accessible to the hydrophilic environment at this step, most likely owing to eversion of the m1A base into the active site of the enzyme. Summarizing the findings, we can conclude that the conformational transitions at steps 2 and 3 reflect the process of damage eversion and incorporation of the intercalating finger Phe-102 into the resulting gap. Nevertheless, it is still difficult to say exactly to which step each event belongs. The fourth and fifth steps of the mechanism represent irreversible hydroxylation of the substrate and disintegration of the substrate–protein complex because the fluorescence of aPu diminishes, pointing to restoration of stacking interactions within the duplex. The values of the substrate hydroxylation step constant (*k_r_*) are consistent with the observed rate constant obtained by PAGE: within the same order of magnitude.

**Scheme 2:**
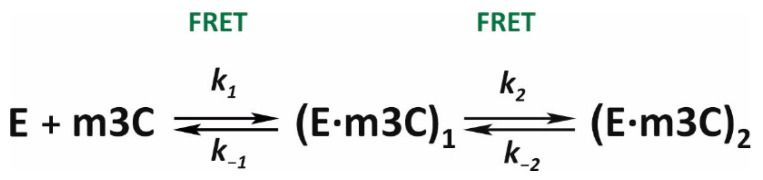
The kinetic mechanism of m3C lesion demethylation by ALKBH2 according to the SF fluorescence data (Figure 5E,F). E is the enzyme coordinated with Fe(II) and 2OG; m3C is a DNA substrate; A is a repaired DNA product; (E∙m3C)_i_ is an intermediate enzyme–substrate complex. Rate constants *k_i_* and *k_−i_* (*i* = 1, 2) characterize forward and reverse directions of the equilibria corresponding to the steps of substrate binding and adjustment for catalysis. The fluorescent probes used for the detection of individual steps are highlighted above the arrows designating the reaction steps.

In this work, conformational dynamics in the ALKBH2/Fe(II)/2OG/m3C complex did not cause any specific shifts in the Trp and aPu fluorescence. The only label sensitive to alterations in the conformation of the enzyme–substrate complex was the FAM/BHQ1 pair, although FRET was not able to detect the step of irreversible hydroxylation of the substrate (Figure 5E). The kinetic curves contained several clearly visible phases of signal change, which lasted for 10 s from the moment of mixing at low enzyme concentrations and up to 50 s at high concentrations (3.0 and 4.0 μM). The results of PAGE analysis (Figure 4) mean that demethylation of the m3C base engaging ALKBH2 requires more time as compared to other tested lesions. Because the estimated half-conversion time for the m3C-containing substrate is 190 s, it can be stated with high confidence that the conformational transitions observed by the SF method correspond to the DNA-binding steps and isomerization of the initial complex but not the step of substrate demethylation. X-ray structures of complexes of ALKBH2 with DNA substrates [17,18] suggest that this enzyme is able to bind various lesions in the active-site pocket. In this context, conversion efficiency of a given substrate depends on how close to the activated metal the oxidized alkyl group is located. It is likely that alkylated analogs of adenine, owing to their larger size as compared to cytosine, can quickly occupy an optimal position deep inside the DNA-binding pocket. The similar shapes of initial parts of the FRET curves obtained for substrates m1A and m3C support this hypothesis. After data fitting, a minimal scheme was determined, which comprises two reversible steps of binding and isomerization of the initial complex (Scheme 2, Table 2).

### 2.5. Conformational Dynamics of εA-Containing DNA during Interactions with ALKBH2

1,N6-Ethenoadenine can simultaneously serve as a DNA lesion and as a fluorescent probe, providing an opportunity for detection of changes in the local environment of the damaged base in the course of interactions with ALKBH2. On the other hand, detection of Trp fluorescence can be impeded by the presence of εA owing to an overlap of their emission and excitation spectra. This is exactly what we observed in preliminary experiments (data not shown). Therefore, in the present study, conformational dynamics of the ALKBH2/Fe(II)/2OG/εA complex were measured by means of the fluorescence of εA and of the FAM/BHQ1 FRET pair.

**Figure 6:**
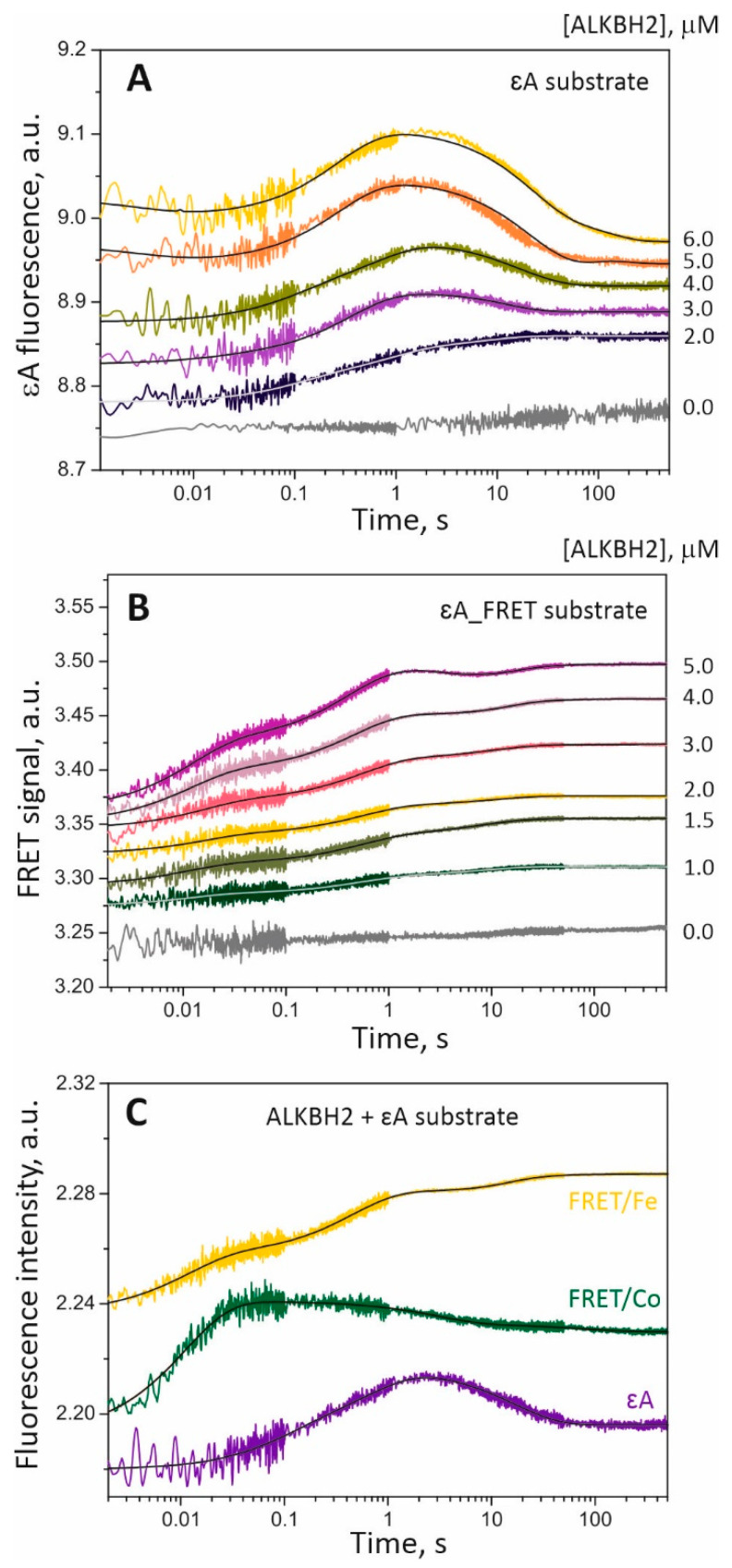
Interactions of ALKBH2 with the εA-containing dsODN as measured by SF analysis. (**A**) Time courses of the εA fluorescence intensity obtained for 3 μM alkylated DNA and various concentrations of the enzyme. (**B**) Time courses of FRET between the fluorophore (FAM) and the quencher (BHQ1) as obtained for 2 μM εA-containing dsODN and various concentrations of ALKBH2. (**C**) Time courses of FRET between the fluorophore (FAM) and the quencher (BHQ1) as determined for 1.5 μM εA-containing dsODN and various concentrations of ALKBH2. Gray curves represent control experiments where the fluorescent component was mixed with the buffer. For each set of curves, jagged traces represent experimental data. Smoothed curves were built via global fitting of the dataset to Scheme 2.

On the SF fluorescence curves obtained at a fixed concentration of εA, several phases of successive changes in fluorescence intensity were observed (Figure 6A). A pronounced increase in the signal, whose amplitude depended on the ALKBH2 concentration, began at 20 to 30 ms and reached a maximum at ~2 s, followed by quenching of the εA fluorescence in the interval between 2 s and 70 s. It is known that the fluorescence of εA and its derivatives is quenched by hydrophobic and stacking interactions and strengthens in a polar environment [41]. In addition, fluorescence intensity of εA in single- and double-stranded oligonucleotides is affected by the nature of the neighboring bases. In particular, purine nucleotides, and especially guanine, that are in the same strand as εA, significantly reduce the fluorescence of the latter owing to strong electrostatic interactions [42]. The model DNA duplexes tested in the present work contained etheno-adenine in the context GεAT, which can strongly quench fluorescence of the substrate. The interaction of substrate εA with ALKBH2 turned out to be accompanied by the eversion of the damaged base from the DNA duplex into the more polar environment of the active site. Accordingly, the increase in fluorescence observed on kinetic curves probably corresponds to the step of εA eversion and its fixation in the active site of the enzyme (Figure 6A). The subsequent signal decline, not detected at low protein concentrations, is most likely driven by the εA-to-A transformation, which is accompanied by the destruction of the fluorophore group. This supposition is supported by the finding that the τ_1/2_ determined by PAGE analysis for substrate εA is 19 s (see Section 2.2), which corresponds to the phase of εA fluorescence quenching on the SF curves.

Changes in the FRET signal describing the conformational dynamics of substrate εA when mixed with ALKBH2 are displayed in Figure 6B. The kinetic curves contain two or three consecutive phases of signal growth (periods 3–70 ms, 0.1–1.0 s, and 10–50 s), whose magnitude depends on the concentration of the enzyme. These transitions may be explained by an alteration in the nature of the interaction between FAM and BHQ1 as a consequence of the DNA substrate helix loosening as well as its bending. In the case of εA, there was no initial drop in the FRET signal on the curves, as documented for the m1A- and m3C-containing substrates above. This finding may indicate differences in structural and dynamic behavior of the system during the initial stages of interaction with damaged bases of different size and structure.

To assess the conformational dynamics of ALKBH2 in the course of binding to substrate εA in the presence of Co(II) ions, the SF kinetic curves were recorded in a buffer containing cobalt instead of iron (Figure 6C, green curve). The change in the FRET signal of the ALKBH2/Co(II)/2OG/εA complex contained one maximum, instead of the two observed for catalytically active complex ALKBH2/Fe(II)/2OG/εA (Figure 6C, yellow curve). Then, the curve for the Co(II)-containing system featured a weak and prolonged decrease in the fluorescent signal, apparently associated with a nonproductive enzyme–substrate interaction. The initial phase of the increase between 2 ms and 30 ms corresponds to the first phase of the increase detected in the presence of Fe(II). Therefore, the growth of the FRET signal in the above period describes the step of initial binding of the enzyme to the DNA.

**Scheme 3:**
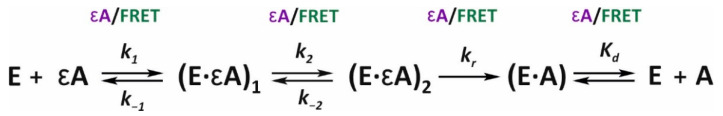
The kinetic mechanism of εA lesion demethylation by ALKBH2 according to the SF fluorescence data (Figure 6A,B). E is the enzyme coordinated with Fe(II) and 2OG; εA is a DNA substrate containing εA; A is a repaired DNA product; (E∙εA)_i_ is an intermediate enzyme–substrate complex. The corresponding kinetic parameters are presented in Table 2.

After the quantitative analysis of the kinetic traces obtained for both types of fluorescent probes (εA and FRET) by the nonlinear-regression method, a four-step molecular-kinetic mechanism was established (Scheme 3). There was good general agreement between the values of kinetic constants obtained using the two fluorophores (Table 2). Thus, the change in the polarity of the εA environment occurred simultaneously with the change in the distance between labels FAM and BHQ1 at each step of interactions with the enzyme.

The initial binding is characterized by almost identical values of rate constants *k*_1_ and *k*_−1_, derived from εA and FRET fluorescence (see Figure 6 and Table 2). After comparing the values of forward and reverse rate constants of the first step among the three lesions (m1A, m3C, and εA), we can conclude that these constants are lower for εA than for the other lesions. Nevertheless, equilibrium association constant *K_a_* = *k*_1_/*k*_−1_, which describes the formation of the bimolecular complex (E∙εA), is 8.6 × 10^5^ M^−1^, which is about twice as high as these values for substrate m1A (3.6 × 10^6^ M^−1^) and substrate m3C (2.3 × 10^5^ M^−1^). Subsequent isomerization of (E∙εA) into a more specific complex, (E∙εA)_1_, also proceeds with high efficiency in terms of the *k*_2_/*k*_−2_ ratio. This process enhances overall affinity of the ALKBH2 active site for εA, as determined by Equation (1):

(1) $$K_{a}^{SF}=\frac{k_{1}}{k_{-1}}\left(1+\frac{k_{2}}{k_{-2}}\left(1+\frac{k_{3}}{k_{-3}}\;\right)\right)$$

This value (*K_a_^SF^*= 1.9 × 10^6^ M^−1^) is 4–5 times higher than this protein’s affinity for m1A (6.0 × 10^5^ M^−1^) and m3C (4.6 × 10^5^ M^−1^). In other words, despite the lower rate of formation of the initial complex between ALKBH2 and εA-containing DNA, the efficiency of this interaction is very high. According to the εA fluorescence data and FRET analysis, *k_r_* (the rate of the irreversible hydroxylation step) is 0.067 and 0.034 s^−1^, respectively, and these values are close to the hydroxylation rate constant obtained for substrate m1A (0.065 s^−1^). The nature of the applied estimation method does not allow one to determine individual rate constants of forward and reverse reactions of the decay of the enzyme’s complex with the reaction product (E∙A), but make it possible to determine their ratio in terms of equilibrium dissociation constant *K_d_*. The *K_d_* values measured by means of εA fluorescence and FRET are 2.2 and 0.4 μM, respectively. This is much lower than this parameter for the ALKBH2 complex with intact DNA containing adenine at the site of damage (7.8 μM). Consequently, we propose that the post-catalytic complex is quite stable and does not decompose immediately.

## 3. Discussion

This work is the first attempt to investigate conformational dynamics of human 2OG-dependent dioxygenase ALKBH2 in the course of DNA methylated substrate recognition and processing by SF fluorescence spectroscopy. By detecting conformational transitions in the enzyme and substrate molecules under pre–steady-state conditions, we were able to evaluate the significance of conformational dynamics for catalysis implementation. Previously, based on the crystal structure of ALKBH2 bound to a DNA substrate, it had been assumed that the binding to the substrate did not induce noticeable conformational rearrangements of the complex owing to the presence of extra loop motifs that grasp both DNA strands and help to identify the lesion by weakened base pairing [17]. Molecular dynamics and QM/MM computations have uncovered the importance of such movements in the protein–DNA complex for the functioning of both EcAlkB and ALKBH2 [21]. Here, we applied various fluorescent probes sensitive to the local environment changes in order to directly examine conformational dynamics in the ALKBH2 protein and its substrate in real time after fast mixing (at time point ~1 ms). The time courses of the fluorescent signal were indicative of substantial changes in hydrophilicity and polarity of the fluorophore environment in the course of enzyme–substrate interaction for each lesion tested (m1A, m3C, and εA). The most pronounced changes detected by means of the fluorescence of Trp, aPu, and FRET signal were observed in the case of demethylation of m1A-containing DNA. Hydroxylation of the other two lesions (m3C and εA) did not cause specific changes of the signal from Trp residues but influenced fluorescence intensity of the probes located in the DNA sequence. Relatively low sensitivity of the Trp residues to the structural dynamics of ALKBH2 can be explained in two ways. Firstly, Trp-105 is probably the only residue within the aa sequence that undergoes a specific change in polarity during the processing of DNA owing to the specific location in the tertiary structure. Thus, opportunities for detecting the conformational dynamics of the protein are limited to the examination of the β3–β4 hairpin. Site-directed mutagenesis of certain residues in the vicinity of the active site can help increase the signal-to-noise ratio of Trp fluorescence during the registration of the enzyme conformational changes. Secondly, ALKBH2 structure may be relatively stable and not change dramatically during catalysis, as previously suggested by Waheed et al. [21]. As concluded by those authors, the DNA substrate contributes substantially to the overall flexibility of the ALKBH2/DNA complex, consistent with our SF data on the dealkylation of substrates m3C and εA.

To date, a significant amount of information has been published about the ALKBH2 activity toward alkylated DNA lesions of different nature [7,8,9,10]. Nonetheless, these studies have been conducted under dissimilar conditions that preclude their comparison. In particular, we could not find any comparison between monoalkyl and etheno lesions in the efficiency of repair by the ALKBH2 dioxygenase under identical conditions. To clarify the effect of different lesions on conformational dynamics, we designed a set of model DNA substrates containing different types of lesion. It was found that under pre–steady-state conditions, the ALKBH2 protein removes lesion m1A from dsDNA with the highest rate and efficiency. Multiple conformational changes in the ALKBH2/Fe(II)/2OG/m1A complex were registered by the SF method, especially by means of a FRET signal. The findings suggest that the double helix of substrate m1A constantly changes its conformation in the course of interaction with ALKBH2, which moves the ends of DNA closer and further apart. The observed transitions reflect a mutual adjustment of enzyme hairpins and DNA grooves to achieve a catalytically active state. Our analysis of Trp and aPu fluorescence traces led to the conclusion that the recognition and binding of DNA by the enzyme at the first moment induces a conformation alteration in the substrate and only then causes changes in the local environment of the Trp-105 residue. The latter changes can be ascribed to the dynamics of the β3–β4 hairpin, which interacts with the damaged DNA strand. Therefore, the recognition of the damaged base within the DNA duplex begins with duplex bending by the enzyme. During monitoring of aPu fluorescence, a local melting of the substrate duplex adjacent to the m1A base was detected right after the conformational transition in the β3–β4 hairpin. We propose that this transition reflects the eversion of m1A into the active-site pocket, followed by its hydroxylation and by restoration of stacking interactions between substrate nucleotides.

Regarding the εA base, its emitted fluorescence offers a great opportunity for researching the repair of this damage by biophysical and spectroscopic methods. A comprehensive computational study [43] on EcAlkb and ALKBH2 preferences for various etheno adducts confirmed the existence of π-stacking interactions between the side chain of His-171 and an alkylated nucleobase. The SF kinetic curves obtained in the present work with the help of εA fluorescence contained clearly distinguishable phases of an increase and decrease, the amplitude of which depends on the enzyme concentration. Moreover, the increase in εA fluorescence takes place soon after the initial binding step and, therefore, may be related to the formation of π-stacking interactions within the ALKBH2 active site. The subsequent quenching of the fluorescence in all likelihood means catalytic oxidation forming a nonfluorescent adenine base. Using these data, the rate of hydroxylation of εA-containing DNA was determined reliably.

The m3C base manifested the lowest demethylation rate, which can be attributed to slow attainment of the catalytically optimal positioning owing to the small size of this base. A detailed crystallographic study performed by the group of Chuan He [18] shed light on the mechanism of the DNA duplex interrogation by ALKBH2. This work revealed the relationship between the stability of different base pairs and efficiency of their recognition and binding by the dioxygenase. It was found that the central C-G pair in the complex ALKBH2-dsDNA is fully intrahelical, albeit the pair is distorted severely, whereas C-I (which has one less hydrogen bond) and A-T pairs display the flipped-out C and A bases, respectively. Moreover, m3C and C were found to be located at the identical spot in ALKBH2 crystal structures complexed to damaged or intact DNA. At the same time, orientation of εA, m1A, and A bases within the active site pocket did not fully coincide. As follows from molecular dynamics simulation, a free energy of breaking the DNA base pair by ALKBH2 decreases in the sequence of C-G > C-I > εA-T. It is very likely that hydroxylation of the m3C base in dsDNA is more challenging for ALKBH2 than transformation of m1A and εA bases. This is also in line with our SF data showing a significant difference in the conformational behavior of the complexes ALKBH2/m1A and ALKBH2/m3C. By analogy with m1A substrate, initial binding of m3C-containing substrate should proceed with high efficiency and demonstrates similar conformational dynamics, as we actually observed using FRET analysis. However, further processing of m3C lesion including base flipping and oxidation requires more energy and time, which was reflected in a slower product accumulation and the absence of sharp conformational changes on the SF curves.

In our previous work, SF spectroscopy was applied for studying conformational dynamics of the EcAlkB/DNA complex by means of lesion m1A as an example [25]. The lengths and nucleotide context of those model single- and double-stranded substrates matched the ones used in the present study. The conformational transitions were also detected via fluorescence of Trp, aPu, and FRET. When shapes of SF curves for the corresponding fluorophores are compared, it becomes clear that the character of signal changes over time differs considerably between EcAlkB and ALKBH2. Particularly, the initial binding to the DNA substrate in the case of EcAlkB causes a notable change in the protein conformation and does not alter the structure of DNA, especially during interactions with the double-stranded substrate. For ALKBH2, we see the opposite: the protein’s domains weakly fluctuate during damaged-DNA recognition and binding. The findings are in good agreement with results of a molecular dynamics simulation [21], which suggest that the structure of EcAlkB is quite flexible, whereas subdomains of ALKBH2 are pre-formed for binding the dsDNA.

In conclusion, our results revealed good prospects for the application of SF spectroscopy to research on enzymatic processes of 2OG dioxygenases. Application of different fluorescent probes to one system can clarify the overall picture of structural dynamics of enzyme–substrate complexes. Transition metals other than iron act as specific inhibitors of the dioxygenase activity without disrupting the ability of this enzyme to recognize and bind alkylated DNA. This approach allows one to distinguish the steps of fine-tuning of the enzyme active site and of its substrate from the catalytic-oxidation step. The detailed insights into structure−function relationships of DNA dioxygenases reveal the crucial elements maintaining DNA integrity.

## 4. Materials and Methods

### 4.1. Preparation of Substrates

Fifteen-nucleotide ODNs were synthesized on an ASM-700 Synthesizer (BIOSSET Ltd., Novosibirsk, Russia) using phosphoramidites purchased from Glen Research (Sterling, VA, USA) and were purified by anion exchange high-performance liquid chromatography. Concentrations of the ODNs were determined via absorption at 260 nm (A_260_). The purity, homogeneity, and integrity of each ODN were assessed by 20% PAGE. The damaged strand 5′-ACAGG**X**TC**Y**GGCATA-3′ contained either m1A or εA at the X position or m3C at the Y position. The complementary strand 5′-TATGCCGGATCCTGT-3′ was annealed to the damaged strand in a 1:1 molar ratio (see Table 1). In certain cases, the damaged strand was modified by FRET labels (6-FAM at the 5′ end and BHQ1 at the 3′ end) or by insertion of an aPu fluorescent base at the seventh position opposite a T base. For PAGE analysis of the enzyme activity, the 6-FAM label was attached to the 5′ end of the damaged strand.

### 4.2. Purification of the ALKBH2 Protein

ALKBH2 was overexpressed in Rosetta 2 cells and purified as described previously [25]. In brief, the enzyme was sequentially purified using a Q-Sepharose Fast Flow column (Cytiva, Uppsala, Sweden) and a HiTrap Ni-chelating column (Cytiva, Uppsala, Sweden). Fractions containing ALKBH2 were pooled and dialyzed against a buffer composed of 20 mM Tris-HCl (pH 7.5), 200 mM NaCl, 2 mM DTT, and 20% glycerol. Finally, the glycerol concentration was brought to 60% (*v*/*v*), and the protein was stored at −20 °C. The protein concentration was determined by the Bradford assay with BSA as a standard.

### 4.3. Equilibrium Fluorescence Spectroscopy

Fluorescence titration of ALKBH2 was performed on a Cary Eclipse fluorescence spectrometer (Varian, Agilent) in a cuvette equilibrated at 25 °C. According to preliminary experiments, the maximum in ALKBH2 fluorescence spectra was 345 nm, and that wavelength was chosen for fluorescence detection. The fluorescence was excited at 280 nm. Assay mixtures of 100 μM apoALKBH2 (free enzyme with no metal or 2OG added), ALKBH2/Fe, ALKBH2/2OG, or ALKBH2/Co/2OG were prepared in a buffer (50 mM HEPES-KOH (pH 7.5), 50 mM KCl, and 10 mM MgCl_2_) and titrated with a metal ion (Fe or Co), 2OG, or an alkylated DNA substrate. The sources of Fe(II) and Co(II) ions were an ammonium iron(II) sulfate hexahydrate [(NH_4_)_2_·Fe(SO_4_)_2_·6H_2_O] and cobalt(II) sulfate heptahydrate [(NH_4_)_2_·Co(SO_4_)_2_·7H_2_O], respectively. The time intervals between additions of a titrant did not exceed 30 s to avoid possible protein aggregation. Fluorescent titration of apoALKBH2 was carried out in the presence of 2 mM EDTA. Each titration curve was the average of three independent experiments. To avoid an undesirable fluorescent signal from εA, a control experiment was conducted, in which the buffer was titrated by the εA-containing substrate. In the figures, for better presentation, the curves were manually moved apart. To determine equilibrium dissociation constant *K_d_*, data were analyzed using Equation (2) in the OriginPro 8.1 software (OriginLab Corp, Northampton, MA, USA).

(2) $$F=F_{0}+f_{2}\times e_{0}+\frac{\left(f_{2}-f_{1}\right)}{2}\left(\sqrt{{\left(K_{d}^{}\;+\;p_{0}\;-\;e_{0}\right)}^{2}+4K_{d}^{}e_{0}}-K_{d}^{}-p_{0}+e_{0}\right)$$

where *F* is the Trp fluorescence intensity measured for each ODN concentration; *F*_0_ is background fluorescence, *f*_1_ and *f*_2_ are partial fluorescence intensities of ALKBH2 with no ODNs added and of an enzyme complex at the saturating ODN concentration, respectively; and *e*_0_ and *p*_0_ are total concentrations of ALKBH2 and ODNs, respectively.

### 4.4. The Restriction Enzyme-Coupled Assay for Direct Dealkylation by ALKBH2

A typical reaction mixture (80 μL) contained 2 μM FAM-labeled DNA substrate and 2 μM ALKBH2 in reaction buffer consisting of 50 mM HEPES-KOH (pH 7.5), 50 mM KCl, 10 mM MgCl_2_, 1 mM 2OG, 2 mM sodium ascorbate, and 40 μM (NH_4_)_2_Fe(SO_4_)_2_·6H_2_O. The reaction was carried out at 25 °C for 120 min and terminated at each time point by the addition of NaOH to 0.1 M and immediate heating to 95 °C. Cooled aliquots were neutralized by HCl and desalted on a MicroSpin G-25 column (Cytiva, Sweden). To visualize the dealkylated DNA product, each sample was next digested with 3 units of restriction endonucleases sensitive to alkylated bases for 1 h at 37 °C. For DNA substrates containing lesion m1A or εA, the DpnII enzyme (recognized sequence: GATC) was applied. The m3C-containing substrate was treated with HpaII (recognized sequence: CCGG). The resulting DNA fragments were separated by denaturing PAGE analysis and visualized with a gel-documenting system, E-box (Vilber, Marne-la-Vallée, France). The data were analyzed under the assumption that a rate constant *k_obs_* was a ratio of the initial velocity, *V*_0_, to the equilibrium concentration of the pre-catalytic complex [ES]_i_ using Equation (3) as described in ref. [25].

(3) $$k_{obs}=2V_{0}{\left(e_{0}+s_{0}+\frac{1}{K_{a}^{SF}}+\sqrt{{\left(e_{0}+s_{0}+\frac{1}{K_{a}^{SF}}\right)}^{2}-4e_{0}s_{0}}\right)}^{-1}$$

where *V*_0_ is the initial velocity calculated from the slope of the linear part of the PAGE time course; *e*_0_ and *s*_0_ are the total concentrations of the enzyme and DNA; and *K_a_* is an equilibrium association constant computed from the values of direct and reverse rate constants of the steps determined in the quantitative analysis of SF data: *K_a_* = *K*_1_ + *K*_1_ × *K*_2_ + *K*_1_ × *K*_2_ × *K*_3_, where *K_i_* = *k_i_*/*k_−i_*, and *i* is an ID number of a step.

### 4.5. SF Kinetics

SF fluorescence curves were acquired at 25 °C on an SX.20 spectrometer (Applied Photophysics, Leatherhead, UK) equipped with a 150 W Xe arc lamp. One syringe contained a fixed concentration (1.5 or 2 μM) of a fluorescent compound (the enzyme or the εA-, aPu-, or FRET-containing DNA substrate) in reaction buffer, and another one contained varying concentrations (0.5 to 5.0 μM) of the nonfluorescent counterpart (the enzyme or the m1A- or m3C-containing DNA substrate) in the same buffer. After rapid mixing of reagents (dead time ~1 ms), a specific fluorescence signal was registered (Table 3), providing a set of kinetic curves for each fluorophore used. To address potential nonspecific changes in the signal, we conducted a set of control SF experiments (data not shown), where the fluorophore-containing component was mixed with the buffer under conditions identical to those of the full system. Typically, each curve represented the average of at least five independent runs. Because changes in the fluorescence signal were detected within a long period (~150 s), each curve was recorded in split mode including the following periods: 1–100 ms, 0.1–1.0 s, 1–50 s, and 50–550 s.

At particular times, different conformational states are present in the bulk solution, and the fluorescence signal is the sum of all fluorescent forms. In the case when concentration of substrate is higher than concentration of enzyme (multiple turnover conditions), the enzyme will interact with new equivalent of substrate and significantly disturb the sequential time distribution of intermediates. Therefore, in the present study, single turnover conditions were used, when concentrations of enzyme and DNA substrate in reaction mixture were near to equimolar. Such experimental conditions facilitated the analysis of experimental data and allowed us to calculate rate constants of intermediates conversion.

To determine the kinetic mechanism and rate constants, the SF data were globally fitted in DynaFit 4 software (BioKin Ltd., Watertown, MA, USA) [39], as described in our previous studies [25,40]. In brief, the SF Trp fluorescence traces were directly fitted to the expression of fluorescence intensity (*F*) at any reaction time point, *t*, as the sum of the background fluorescence (*F_b_*) and fluorescence intensities of all protein species (Equations (4) and (5)),

(4) $$F=F_{b}+\overset{n}{\underset{i=0}{\sum }}F_{i}\left(t\right)$$

(5) $$F_{i}\left(t\right)=f_{i}\times \left[E_{i}\left(t\right)\right]$$

where *f_i_* is the coefficient of the specific fluorescence for each discernible ALKBH2 conformer, and [*E_i_*(*t*)] is the concentration of the conformer at any given time point *t* (*i* = 0 corresponds to the free protein, and *i* > 0 to protein–DNA complexes). These specific fluorescence coefficients described only the part of the fluorescence that changed due to the DNA binding. The software performed numerical integration of a system of ordinary differential equations with subsequent non-linear least-squares regression analysis. Similar fitting procedures were carried out for aPu fluorescence and FRET signal, except that fluorescently discernible transient states corresponded to DNA species. Error in all rate constant values was determined as the standard deviation of kinetic constants derived from different fitting iterations.

## Figures and Tables

**Table 1:** The structure of double-stranded oligodeoxyribonucleotides (dsODNs) used as substrates for ALKBH2. m1A: N1-methyladenine; m3C: N3-methylcytosine; εA: 1,N6-ethenoadenine.

Shorthand	Duplex Structure
m1A	5′-ACAGG**m1A**TCCGGCATA-3′ TGTCCTAGGCCGTAT
m1A_aPu	5′-ACAGG**m1AaPu**CCGGCATA-3′ TGTCCTTGGCCGTAT
m1A_FRET	5′-**FAM**-ACAGG**m1A**TCCGGCATA-**BHQ1**-3′ TGTCCTAGGCCGTAT
m3C	5′-ACAGGATC**m3C**GGCATA-3′ TGTCCTAGGCCGTAT
m3C_FRET	5′-**FAM**-ACAGGATC**m3C**GGCATA-**BHQ1**-3′ TGTCCTAGGCCGTAT
εA	5′-ACAGG**εA**TCCGGCATA-3′ TGTCCTAGGCCGTAT
εA_FRET	5′-**FAM**-ACAGG**εA**TCCGGCATA-**BHQ1**-3′ TGTCCTAGGCCGTAT

**Table 2:** A summary of kinetic parameters for DNA dealkylation by the ALKBH2 protein, as determined by global fitting.

Lesion	m1A			εA		m3C
Type of Probe	Trp	aPu	FRET	εA	FRET	FRET
*k*_1_ × 10^−6^, M^−1^ s^−1^	7.3 ± 1.7	0.70 ± 0.16	115 ± 18	47 ± 10	48 ± 3	52 ± 13
*k*_-1_, s^−1^	123 ± 16	3.4 ± 0.6	319 ± 63	63 ± 9	56 ± 4	230 ± 26
*k*_2_, s^−1^	7.2 ± 0.9		10 ± 3	1.9 ± 0.2	2.0 ± 0.1	0.45 ± 0.22
*k*_-2_, s^−1^	3.2 ± 0.3		21 ± 8	1.26 ± 0.09	1.74 ± 0.07	31 ± 8
*k*_3_, s^−1^	0.89 ± 0.03	0.67 ± 0.13	2.2 ± 0.2			
*k*_-3_, s^−1^	1.3 ± 0.1	0.07 ± 0.05	6.1 ± 0.7			
*k_r_*, s^−1^		0.04 ± 0.01	0.065 ± 0.012	0.067 ± 0.010	0.034 ± 0.005	
*K_d_* × 10^6^, M		27 ± 2	1.4 ± 0.2	2.2 ± 0.1	0.4 ± 0.01	

**Table 3:** A summary of fluorescent probes used in the study.

Fluorophore	Excitation Max., nm	Emission Max., nm	Filter Type
Trp residues of ALKBH2	300	345	WG-320
εA	316	410	LG-370
aPu	310	360	LG-370
FAM-BHQ1 pair	494	530	OG-530

## Sample Availability

Samples of the compounds are not available from the authors.

## Abbreviations

ALKBHi, human homologs of AlkB from *E. coli*; aPu, aminopurine; 2OG, 2-oxoglutarate; BHQ1, black hole quencher; dsDNA, double-stranded DNA; dsODN, double-stranded oligodeoxyribonucleotide; εA, 1,N6-ethenoadenine; 6-FAM, 6-carboxyfluorescein; EcAlkB, *E. coli* AlkB; FRET, Förster resonance energy transfer; m1A, N1-methyladenine; m3C, N3-methylcytosine; ODN, oligodeoxyribonucleotide; PAGE, polyacrylamide gel electrophoresis; SF, stopped-flow.
